# Re-evaluation of the myoepithelial cells roles in the breast cancer progression

**DOI:** 10.1186/s12935-022-02829-y

**Published:** 2022-12-12

**Authors:** Anwar Shams

**Affiliations:** Department of Pharmacology, College of Medicine, Taif University, P.O. BOX 11099, Taif, 21944 Saudi Arabia

**Keywords:** Myoepithelial cells, Breast cancer, Polarity, Invasion, Differentiation

## Abstract

Over the past decades, luminal epithelial cell lineage has gained considerable attraction as the functionally milk-secreting units and as the most fruitful acreage for breast cancer launching. Recognition of the effective involvement of the myoepithelial cells in mammary gland development and in hampering tumorigenesis has renewed the interest in investigating the biological roles of this second main mammary lineage. The human breast is made up of an extensively branching ductal system intervening by copious lobular units. The ductal system is coated by a chain of luminal epithelial cells (LECs) situated on a layer of myoepithelial cells (MECs) and encompassed by a distinguished basement membrane. Ductal contractility during lactation is a well-known function delivered by the MECs however this is not the only assignment mediated by these cellular populations. It has been well appreciated that the MECs exhibit a natural paracrine power in defeating cancer development and advancement. MECs were found to express numerous proteinase inhibitors, anti-angiogenic factors, and tumour suppressors proteins. Additionally, MECs contributed effectively to maintaining the right luminal cells' polarization and further separating them from the adjacent stroma by making an integrated fence. Indeed, disruption of the MECs layer was reported to facilitate the invasion of the cancer cells to the surrounding stroma. Nonetheless, MECs were also found to exhibit cancer-promoting effects and provoke tumour invasion and dissemination by displaying distinct cancer chemokines. Herein in this review, we aimed to address the roles delivered by MECs in breast cancer progression and decipher the molecular mechanisms regulating proper MECs’ physiology, integrity, and terminal differentiation.

**Graphical Abstract:**

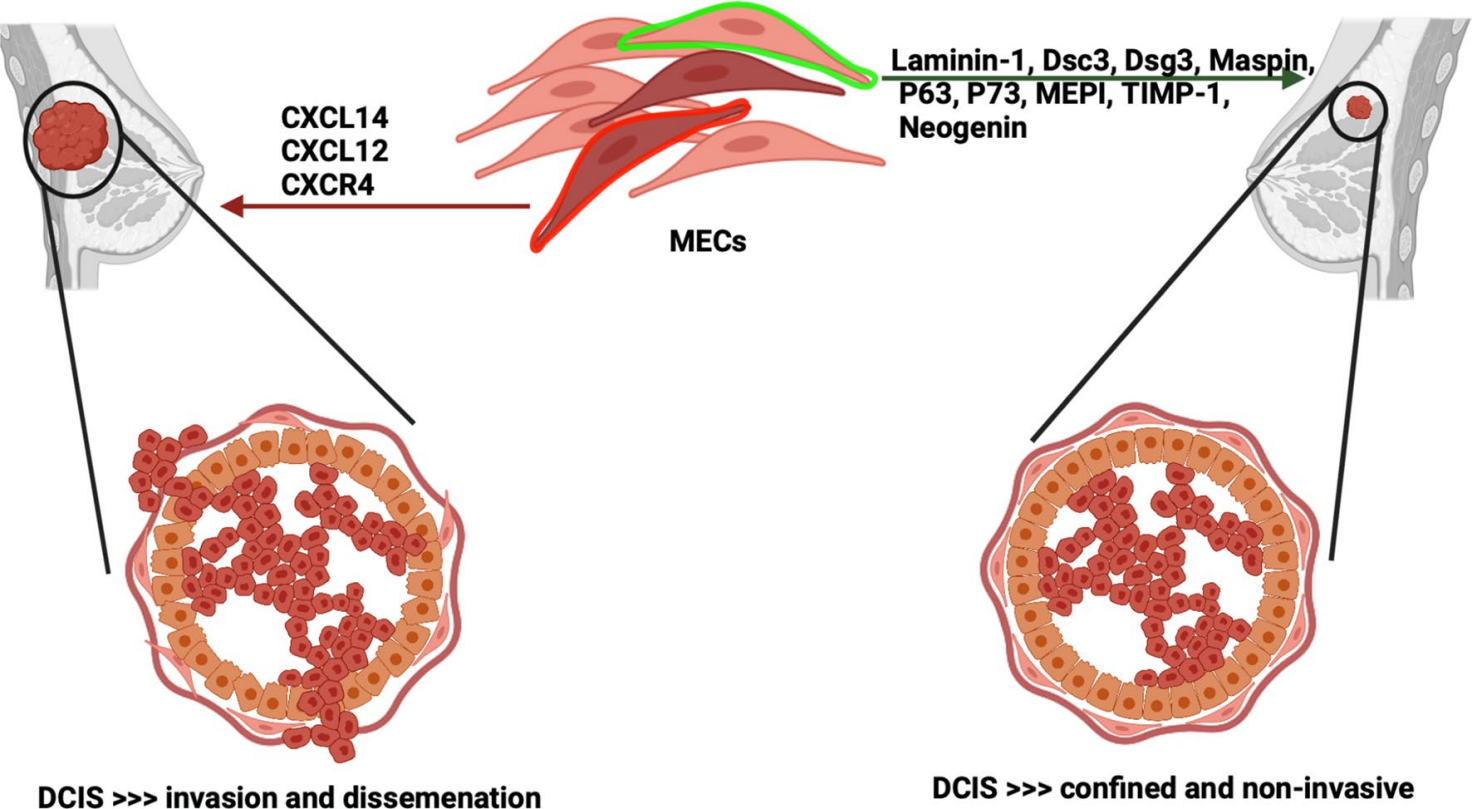

## Introduction

The mammary gland is an exocrine organ and a supplementary reproductive structure providing the premise nutritional source for postpartum life [1]. The mammary gland is composed of numerous elongated ductal branches embedding in a background of fatty tissues. Two major cell types cooperate to build up the ductal units: inner polarized luminal epithelial cells (LECs) embraced by a layer of myoepithelial cells (MECs), both cell types are rimmed by a cohesive basement membrane (BM) [2]. The LECs have attained most of the investigators’ attraction for prolonged eras as they are deemed to be the central origin of breast cancer evolution. Subsequently, a plethora of studies have been conducted to elucidate the physiological functions and pathological conditions associated with LECs while the MECs were left undervalued [3]. Hitherto the involvement of the MECs in mammary gland orchestration and morphogenesis [4] and in affording fortification against tumour progression and invasion [5, 6] has re-established the attention in studying and characterizing the MECs.

Different growth factors, chemical compounds, and tumour epithelial cells must first pass through both layers of the MECs and the BM to connect with the surrounding stroma; as a result, both layers work together to function as reliable gatekeepers to the outside world [3]. Subsequent in vitro and in vivo studies have demonstrated the initial steps of cancer cell invasion and dissemination by disrupting the integrity of the myoepithelial belt that encircles the LECs [7–11]. Therefore, damaging the MECs layer resulted in the release of various factors (such as SDF1/CXCL12, CXCL14, MMP, and tenascin) [11, 12] with a potential to modify the tumour microenvironment and facilitate the paracrine communication between the tumour epithelial cells and the enclosed stroma enhancing the tumour aggressiveness [11, 13]. Moreover, efforts have been engendered to study the relationships between the MECs and the LECs and their engagement in breast cancer tumorigenesis and plasticity. Accordingly, another area of research about how the LECs and MECs originate and molecularly communicate in both normal and pathological conditions was also investigated [14].

In this review, we provided an overview of the myoepithelial cells’ histogenesis, molecular and biological markers, and physiological commitments. Next, we re-evaluate the important role of MECs in breast cancer evolution and progression. Finally, we shed the light on the key molecular signalling pathways that are involved in the regulation of MECs' development and functions. Identifying the precise mechanisms by which MECs battle against tumorigenesis will provide further guidance on the decision-making implicating therapeutic options for breast cancer patients.

## Anatomical and histogenesis portrayal of the myoepithelial cells

**Fig. 1:**
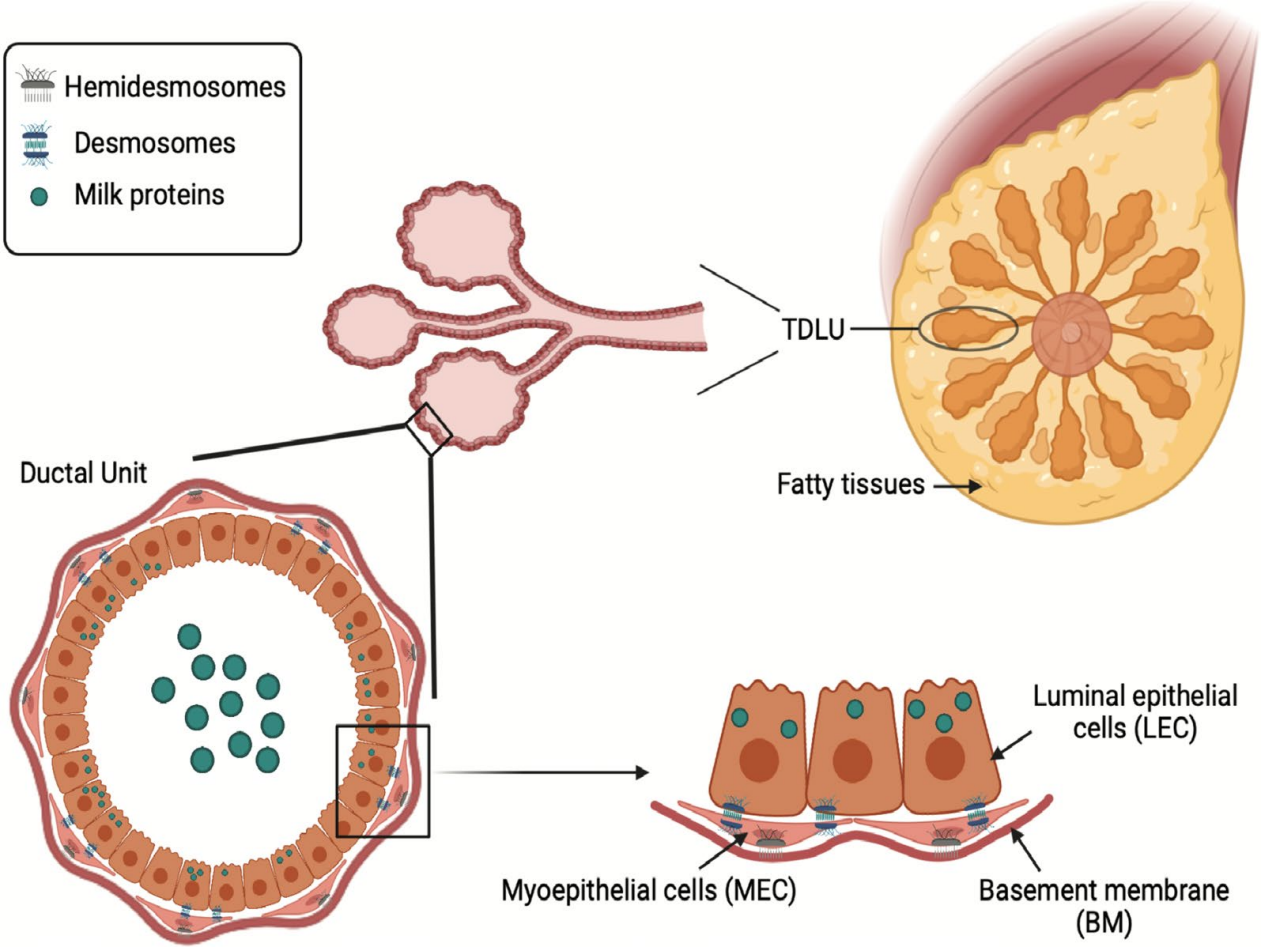
Schematic presentation of the main morphological aspects of the mammary gland: anatomical structure of the normal mammary gland composed of numerous terminal ductal lobular units (TDLUs) embedded in the parenchyma of fatty tissues. A cross-section illustration of the ductal bilayer unit made of the inner layer of the polarized secretory luminal epithelial cells (LECs) and the outer layer of myoepithelial cells (MECs), all enclosed within the basement membrane (BM). The LECs secreted milk proteins during lactation. The MECs are connected to the LECs by desmosomes and the underlying BM by hemidesmosomes. Created with BioRender.com and adapted from the following references: [15, 16]

We can better grasp a variety of breast diseases, including breast malignancies if we have a better understanding of the mammary gland's normal morphology [15]. The human breast is the apocrine milk-producing gland the primary source of substance for infants. The main unit of the mammary gland consists of copious terminal ducts lobular units (TDLU) that branched throughout the fatty tissues of the breast parenchyma, (Fig. 1). TDLU, as implied by its name, consists of ductal branching networks and lobular structures containing milk-secreting acini [4, 16].

The ductal unit is organized into a bi-layer system of LECs lining the inner surface and distributed in a polarized fashion with an apical side facing the lumen and basal bottom situated on a layer of MECs [4]. MECs are specialized cells that combine the phenotypic features of both epithelial cells and smooth muscle cells. MECs display the expression of smooth muscle markers (Myo-) such as actin, myosin, and vimentin filaments and exhibit a contractile activity. Additionally, MECs are akin to the epithelial phenotype by showing the expression of different epithelial molecular markers (-epithelial) mainly cytokeratins [17]. Other epithelial proteins include desmosomes, hemidesmosomes, and cadherins (P-cadherin) with other various cellular junction proteins [2, 4]. Cadherins are adherents’ junctional proteins that are key mediators in maintaining accurate cellular communications and thus ensuring proper signalling interactions to preserve normal tissue development and morphology. P-cadherin in the mammary gland is exclusively expressed by the MECs that line both ductal and alveolar compartments. Lack of P-cadherin expression in animal models was found to be associated with premature lobular-alveolar formation. The mammary gland of the virgin P-cadherin deficient mice showed extensive ductal tree branching and acini-like structures bestowing features of lactogenic differentiation that mirror a pregnancy background. Moreover, these P-cadherin deficient mice, at the adult stage, displayed aberrant hyperplastic and dysplastic manifestations [18]. Thus suggesting that perturbances in the gene expression pattern by MECs could eventually affect the morphogenesis of the epithelial compartment within the breast tissues [3].

In TDLU, MECs have organized into spindle-shaped cells as a continuous belt lining the ductal unit while in the lobular compartment they are forming a discontinuous layer of stellate-shaped cells surrounding the acini [14]. As shown in Fig. 1, this ductal structure (LECs and MECs) of the mammary gland is extensively branching and confined by a distinguish BM surrounded by a stomal compartment [4, 19]. Because of the direct communication of MECs with the BM, MECs showed a high expression level of different proteins that are also expressed by BM such as integrin dimers, collagen receptors a1b1and a2b1, fibronectin receptor a5b1, and a_v_b3 integrin [20, 21]. Additionally, fully differentiated MECs synthesize numerous components that play role in sculpting ductal elongation and morphogenesis, and in the production of new BM, these include collagen IV, integrin, fibronectin, nidogen, matrix metalloproteinases (MMPs) such as MMP2 and MMP3 [22], morphogens, various growth factors, desmosomes, high level of laminin-1 and 5, and laminin receptors a3b1, a6b1 and a6b4 [15].

## Putative myoepithelial cells progenitors at a glance

**Fig. 2:**
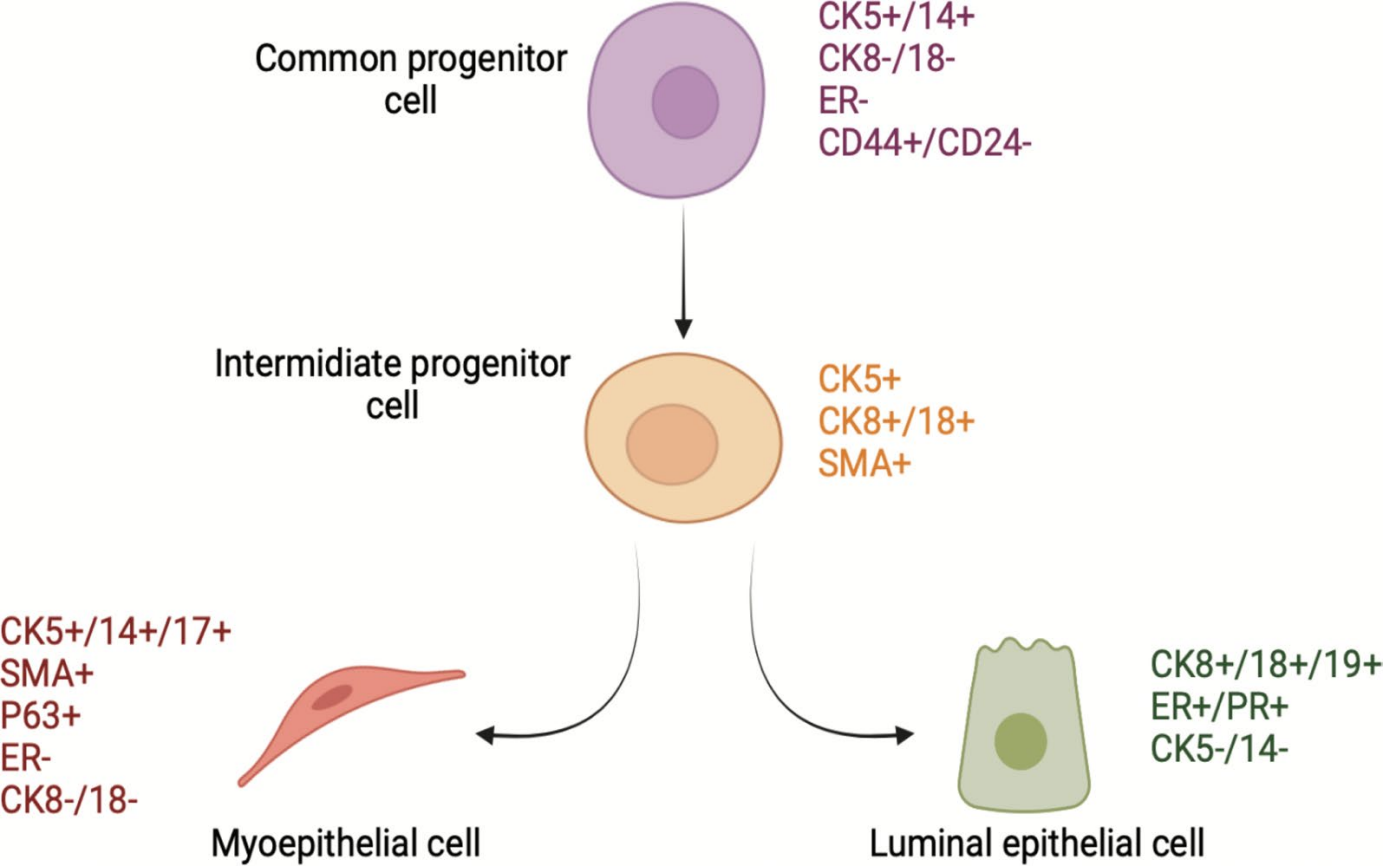
Schematic presentation of the basic mammary stem cells hierarchy: common stem cell progenitors give rise to the intermediate progenitors that terminally differentiated into luminal epithelial cells and myoepithelial cells lineages. Specific molecular marker expressions distinguish each cellular progeny. *α-SMA* α-smooth muscle actin, *Ck* cytokeratin, *ER* estrogen receptor, and *PR* progesterone receptor. Created with BioRender.com and adapted from the following reference: [126]

Both mammary LECs and MECs originated from the ectoderm, unlike the smooth muscle cells that derived from mesoderm and neural crest cells [18]. In the epithelium of the human breast, common putative mammary stem cell ancestors are located within the luminal epithelial components harbouring the expression of CK5 + , (Fig. 2). These progenitors can ultimately differentiate into either the LECs (CK8/18 +) or the MECs (SMA +) after passing with the intermediary differentiated phase (CK5 + and CK8/18 + or SMA +) cells. Using specific antibodies against different molecular markers, the LECs were further characterized by the expression of CKs 8/18/19 and estrogen/progesterone receptors while the MECs express CKs 5/14 and SMA [23]. Different basal cytokeratins such as CK 5, CK 14, and CK 17 that are expressed by the basal cells were found to be also expressed by the MECs. These CKs, particularly CK 5 and CK 14, regulate the connection of MECs to underlying BM through hemidesmosome proteins and connect MECs to the adjacent LECs via desmosome proteins, as demonstrated by Fig. 1 [14]. Furthermore, MECs express abundant levels of integrins and adherent junction proteins such as vinculin, talin, focal adhesion kinase, and alpha-actinin that connect LECs to the BM [3]. These anatomical connections accelerate exchanging of many paracrine signalling within the ductal bi-layer units [15].

## Designations of the myoepithelial cells’ physiological duties

MECs have been reported to regulate a spectrum of physiological functions within the mammary gland mainly ductal contraction to release milk during lactation [15]. The lactation process is achieved by secreting the milk into the lumen by the LECs and then ejection of the milk is made by the contractile effects of the MECs in response to pulsatile stimulation of oxytocin from the pituitary gland. Next, Oxytocin interacts with G protein-coupled receptor on the MECs surface and induces a contraction [19]. Fully differentiated MECs and their ultrastructure are characterized by the presence of a large amount of *α*-smooth muscle actin (*α*-SMA) and heavy chain-myosin (hc-myosin) filaments [3]. These filaments are enriched in the reminiscent muscle specific-cytoskeleton and contractile proteins to stimulate the propelling of the milk release to the exterior during the lactation [24]. Both in vivo and in vitro studies found that loss of *α*-SMA expression caused impairment of the contractile activity of MECs and failure of milk ejection and lactation while the mammary gland still presented normal architectures [25]. Additionally, the function of MECs is more than contractility, MECs control the exchange of the physiological signals between the LECs and the BM [15]. MECs by separating the abnormally dividing cells from invading the surrounding stroma/parenchyma form a protective physical and molecular boundary [26]. Likewise, MECs are heavily engaged in maintaining organ homeostasis and structural integrity by orchestrating the LECs into an accurate polarization [15].

## Do we still ruminate myoepithelial cells as an effective barrier against breast cancer progression?

### A. Myoepithelial cells layer as a protector fence against tumour dissemination

It should be born in mind that MECs by forming an integrated layer that separates the LECs from the surrounding stroma provides a natural paracrine defence mechanism against cancer invasion and metastasis [27, 28]. Moreover, MECs by maintaining proper organization and cellular polarity provides extra protective power against the malignant transformation [3, 29]. Indeed, loss or interruption of the fully differentiated MECs layer continuity has been observed during breast cancer progression and was found to accelerate the microinvasion of the cancer cells into the neighbouring stroma [30]. This damage to MECs could have resulted from different factors including mechanical injury, immune infiltration, or loss of cellular repair capacity [13]. Furthermore, both in vitro and in vivo reports have shown the tumour growth and invasion inhibitory effects that are delivered by MECs thus suggesting a safeguarding role exhibited by this population [5, 28]. Therefore, the acknowledgment of the engagement of MECs in controlling breast morphogenesis and cellular polarity to shield against cancer progression has raised the interest in investigating the MECs biology [14, 28].

A surrogate marker used by pathologists in distinguishing between ductal carcinoma in situ (DCIS) and invasive breast cancer (IBC) is the detection of an intact functional MECs layer that rims the LECs [29]. DCIS is the earliest non-invasive form of breast cancer manifested by the presence of an undamaged ring of MECs surrounding hyperproliferative neoplastic luminal cells [31, 32]. MECs bound the DCIS either in a continuous layer or in focally disrupted loci yet both conditions show a similar pattern of immunoreactivity to different molecules (such as maspin and TIMP-1) akin to the normal MECs [33]. Retaining functional intact MECs layer encircling DCIS may alter the tumour evolution and malignant transformation restricting the conversion from precancerous DCIS into invasive ductal carcinoma [34]. Nevertheless, contrary to what has long been thought, lack of MECs layer does not always denote invasive lesions. Indeed, in some breast cancer cases, the absence or focal loss of MECs ring is a feature of an aberrant infiltrative growth pattern yet not adequate to persuade malignancy or invasiveness. For example, Micro-Glandular Adenosis (MGA), infiltrating epitheliosis, fibroadenoma, and apocrine lesions are rare non-invasive breast tumours characterized by the absence of MECs layer and infiltrative cellular proliferation [35].

Accordingly, it was recently discovered that interruption of the MECs layer surrounding DCIS lesions was associated with a lower chance of developing invasive breast cancer as well as a lower rate of cancer recurrence [36]. Using multiplexed ion beam imaging by the time of flight (MIBI-TOF) and 37-plex different antibody staining profiles, a recent comparative study was conducted to compare the gene expression profiles of 79 surgically resected samples of normal breast tissues with matched DCIS non-progressors and DCIS progressors (i.e., IBC) tumours. Surprisingly, samples of DCIS that have progressed and still have the MECs rim intact revealed higher E-cadherin expression as well as a higher risk of invasion and recurrence. DCIS-non progressor samples, however, showed low E-cadherin levels, thinner and disrupted MEC layer, and remained locally confined. Moreover, DCIS (progressors) showed structural and phenotypic features in a similar trend to normal breast tissue. These findings could be explained that the incomplete layer of MECs delivers a defence mechanism against cancer progression by enabling the influx of the immune cells from the reactive stroma into the tumour cells encouraging stromal sensing of the tumour [36]. Indeed, relative to normal tissues and DCIS (progressors) there was an infiltration of stromal mast cells and CD4 T-cells into DCIS (non-progressors) while the stroma of DCIS (progressors) showed enrichment of proliferating cancer-associated fibroblasts (CAFs) and collagen fibres [37]. These data provide substantial insight into the potent influences of the tumour microenvironment and surrounding stroma on breast cancer progression and could stratify the patients into high/low-risk groups for the probability of tumour invasion and recurrence.

### B. Myoepithelial cells as regulators of cellular polarity

The normal ductal system lined by polarized and organized LECs is surrounded by a layer of MECs encompassing integrated BM. This anatomical structure along with the apical-basal polarity of the LECs delivered a natural immunity against breast cancer development [38]. Disturbance of luminal epithelial cells’ polarity and organization was reported as an early step in the breast cancer growth [39]. Moreover, loss of polarity genes such as Par3 and LKB1 was associated with highly invasive breast cancer and poor patient outcomes [40–42].

Among various molecules secreted by MECs, are laminin-1 and desmosomal proteins account for essential mediators in regulating the luminal cells' function, morphology, and polarization [15]. Indeed, laminin-1 expression ensures accurate LECs polarization that is cultured in a 3D collagen system [43, 44]. Culturing of LECs in 3D collagen I gel (lacking laminin) resulted in acini formation with reverse polarity. On the other hand, LECs cultured in a laminin-rich extracellular matrix (lrECM) produced well-polarized acini with a basal pole facing the BM. These findings stress that LECs rely completely on the myoepithelial cells produced laminins to orchestrate them into the polarized fashion [43]. Moreover, the inability to produce laminin-1by the cancer MECs in breast cancers failed transmission of necessary signals and cues to maintain LECs in baso-apical polarity and organization [3, 43].

likewise, desmosomal proteins were reported to contribute significantly to the generation of acinar-like structures. Desmosomes were found to contribute to the connection between the two layers of the mammary epithelium [3]. Consequently, suppression of specific desmosomal cadherins produced exclusively by MECs such as desmocollin 3 (Dsc 3) and desmoglein 3 (Dsg 3) caused disturbed morphology and arbitrary structures by blocking the formation of bilayer acinus unit [45, 46]. An elegant study demonstrated the key role mediated by desmosomes in maintaining the proper organization of the ductal bi-layer system within the mammary gland. Using specific peptides targeting the desmosomal cadherins yielded in ablation of alveologenesis, ductal tree formation, and rightful place alignment [3]. These results validated the indispensable role of desmosomes, produced by MECs, in inducing an accurate cell positioning [3, 45, 46].

### C. Myoepithelial cells safeguard against cancer advancement: angiogenesis, invasion, and proliferation

The main factor causing cancer morbidity and mortality is the spread of cancer cells. The local invasion of the tumours into the surrounding stroma was the first step in the metastatic process. The invasion of the blood vessels that follow, travelling through the circulation, finally culminates in the extravasation of the cancer cells and their invasion of the new location. Certain characteristics must be present in cancer cells for them to survive the multi-step metastasis process and remain in the target organs [47, 48]. The ability of cancer cells to dissolve the BM and enter the surrounding stroma by expressing proteolytic enzyme activities such as those of the MMPs family and plasminogen activation system (PAS) is one of the key dogmas for their invasion and metastasis [48]. Inhibition of proteolytic enzyme activity has demonstrated potential encouraging outcomes in preclinical studies [8, 47, 49]. Furthermore, neovascularization or angiogenesis is an important element in cancer metastasis and spread. Many angiogenic factors have been recognized as potent contributors to breast cancer growth and progression including IL-8, VEGF-A, and MMP-9 [50, 51]. Blocking of angiogenesis has shown fruitful suppression effects of tumour growth, invasion, and migration. Therefore several anti-angiogenic drugs have been used such as biological factors that target the expression of the vascular endothelial growth factor (VEGF) [52].

The MECs layer that surrounds the DCIS, possesses an endogenous resistance to transformation, therefore, tumours of MECs, when it happens, it is usually of low-grade malignancy [5, 43]. An exception is the malignant myoepithelioma which is an extremely rare case of breast cancers [53]. Low invasion lesions of MECs can be attributed to a high level of DNA repair capability and the expression of high levels of proteinase inhibitors and antiangiogenic factors [6, 54–56]. Barsky et al. was the pioneer in describing the antitumorigenic capacity of MECs via suppression of tumour invasion and inhibition of the angiogenesis [56, 57]. In vivo investigations also showed the anti-invasion properties exhibited by MECs in suppressing breast cancer advancement due to the high expression of serine proteinase inhibitors [27]. Moreover, the acknowledgment of the endogenous power in defeating tumour growth and spread owned by MECs can be credited to maspin secretion by these cells [2, 58]. Maspin belongs to the serine proteinase family of inhibitors and it is known for its antitumorigenic activity, suppression of cellular migration, tumour invasion and dissemination, and harbours angiogenesis diminishing effects [44]. MECs along with other normal cells revealed a high expression level of maspin on the contrary maspin expression is largely downregulated in tumour cells highlighting the strong tumour suppression function produced by the maspin [59]. Indeed, the MDA-MB-453 breast cancer cell line that showed overexpression of maspin displayed a less aggressive phenotype featured by the low growth rate, reduced angiogenic, and invasion capacities [44]. In addition to maspin, several tumour suppressor proteins were found to be expressed by specifically MECs including 14–3-3 sigma, p63, p73, and Wilms Tumor [60–62].

The lessening of cell growth and proliferation and reduction of the metastatic capacity in the MCF-7 breast cancer cell line were observed in response to nitric oxide (NO) production. Relaxin, a peptide expressed by the MECs, was found to enhance NO production in many cell types including breast cancer cells hence another evidence of the endogenous tumour beating power exhibited by MECs [63]. MECs were previously reported to produce several proteins that are involved in the synthesis and maintenance of ECM and BM including collagens, laminin, fibronectin, osteonectin, and Thrombospondin-1 [6, 54–56]. One of the major components of the ECM is Thrombospondin-1 (TSP-1) a 340-kD glycoprotein that is secreted by MECs and found to weaken angiogenesis both in vitro and in vivo studies [2]. Breast cancer progression and spread were also found to be overcome by myoepithelium-derived serine proteinase inhibitor (MEPI), a protein that is exclusively expressed in normal and non-invasive MECs. In vivo model showed that expression of MEPI was associated with a reduction in tumour proliferative profile and blocking of invasion capacity and metastasis [64]. Another specialized structure expressed by cells located in the terminal end buds (TEBs) of the mammary gland is the neogenic receptor. Indeed, the cap cells of the TEB differentiate eventually into myoepithelial cells thus implying the expression of neogenin by MECs [3]. Neogenin identified as a member of the NCAM family plays a role in maintaining cellular adhesion and neuronal guidance. Expression of neogenin was reported by Lee et al. to be negatively correlated with breast cancer progression and evolution [65].

The ability of MECs to inhibit the tumour cells invasion was also attained by attenuating the expression of MMPs produced by tumour cells and fibroblasts [22]. TIMP-1 belongs to a family of tissue inhibitors of metalloproteinases (TIMPs) that consists of four members: TIMP1, TIMP2, TIMP3, and TIMP4. TIMP1 is constructively and specifically secreted by MECs and found to contribute to the inhibition of the MMPs expression and neovascularization [33, 66]. Moreover, the contribution of intact MECs layer in limiting the migration and invasion capacities of the luminal cancer cells was investigated in a 3D organoid culture assay using cell linage with Twist1 overexpression, the prometastatic transcription factor. In this model, MECs formed a barrier that confines the Twist1 overexpression luminal cells and detains their local invasion and spread to ECM [67]. The author attributed the MECs' anti-invasive effect to their contractility and adhesive features manifested by the expression of SMA and P-cadherin that promote luminal-myoepithelial cells' effective interaction. Loss of myoepithelial SMA or P-cadherins compromised the ability of the MECs barrier to restrain the escaping invasive cancer cells in the 3D culture [67–69]. Another 3D culture model has scrutinized and emphasized the anti-invasive property of the intact MECs mediated by CPEB1 expression. CPEB1 is a sequence-specific RNA-binding protein that is predominantly produced by MECs during cellular differentiation. Loss of CPEB1 expression increases the mRNA levels of ZEB1, N-cadherin, vimentin, and Twist1 while downregulating the E-cadherin expression, facilitates epithelial-to-mesenchymal transition (EMT), and suppresses the p63 expression in MECs. Furthermore, CPEB1-silenced cells exhibited unorganized non-polarized proliferating colonies with invasive margins expanding through the ECM in the 3D culture system [70].

In comparison to non-myoepithelial cell lines, the myoepithelial cell lines HMS-1–6 (originated from benign myoepithelial tumours), demonstrated a higher expression level of proteinase inhibitors [5]. Also, HMS-1–6 cells are highly enriched in diverse angiogenic inhibitors including TIMP-1, maspin, thrombospondin-1, and bFGF receptors while they displayed low expression levels of angiogenic factors such as bFGF, VEGF, TFGα, TGFβ, HB-EGF, and PD-ECGF [5]. Indeed, exposing both cell lines to a hypoxic environment resulted in upregulation of HIF-1α yet the low expression of VEGF or iNOS was only observed in myoepithelial cells (HMS-1–6) in contrast to non-myoepithelial cells line. This suggests the MECs try to maintain a lower level of angiogenic stimulants as compared to carcinoma cells in response to low O2 tension. Furthermore, the xenograft model of the myoepithelial cell line (HMS-X-6X) revealed low proliferative capacity and is devoid of angiogenic factors as compared to the non-myoepithelial carcinoma counterpart. This model (HMS-X-6X) also showed an abundant ECM that enriches angiogenic inhibitors and exhibits a metastatic suppression effect [33]. Accordingly, the highly metastatic *neo*C8161 cells were injected in both xenografts models resulting in diffuse metastatic niches in the non-myoepithelial model associated with pulmonary infiltration. On the hand, the myoepithelial xenograft presented only a locally restricted area of invasion around the injection site with a complete absence of pulmonary metastasis. These results further authenticated the anti-invasion/anti-metastatic power delivered by MECs. These potent tumour suppressor effects can be ascribed to the presence of an abundance of anti-angiogenic factors (maspin, thrombospondin-1, TIMP-1, soluble bFGF receptors, and prolactin and plasminogen fragments) that are found in the myoepithelial xenograft extracts which play an essential role in shielding against cancer dissemination [33].

To frame the picture, a global gene expression comparative analysis of 22,000 genes using microarray Gene Chips was conducted to compare the genetic profile of the myoepithelial cell lines (HMS-1–6) and its xenograft model with the non-myoepithelial breast cancer cells including MDA-MB-231, MDA-MB-468, inflammatory breast carcinoma samples, normal mammary epithelial cell line (HMEC), and normal breast tissues. The outcome of this study characterized the myoepithelial cells line and its xenograft with a distinguished profile manifested by increased expression of diverse genes related to anti-angiogenic factors (thrombospondin-1 and plasminogen) and proteinase inhibitors (maspin and PAI-1) and ECM proteins (collagens, laminin, fibronectin, and osteonectin) as compared to the other examined clusters [33].

Besides the role of MECs in protecting against neovascularization, invasion and metastasis, their contribution to cellular growth suppression was also reported. Many reports have shown the effect of myoepithelial-conditioned media on inhibiting breast cancer cell proliferation and provoking cell cycle arrest at the G2/M phase [64, 71, 72]. Collectively, these findings identified the MECs as multi-potent fighters by possessing various defending molecular strategies in fighting neoplasms. Likewise, these data established and validated the solid and officious involvement of the MECs in combating breast cancer tumorigenesis and growth, invasion and spread, and angiogenic capacities.

### D. Myoepithelial cells’ cancer-promoting effects

Nevertheless, in some breast cancer cases, MECs were reported to encourage tumour development rather than beating against cancer advancement via the expression of different chemokines. Chemokines and their associated receptors were reported to have a distinctive impact on tumour development and progression [73]. Cancer myoepithelial cells and myofibroblasts that surrounded DCIS exhibited an altered genetic profile characterized by high expression levels of CXCL14 and CXCL12 chemokines respectively as compared to normal intact MECs [74]. These chemokines interact with the epithelial receptors promoting tumour growth and inducing an aggressive phenotype following both paracrine and autocrine pattern [75]. Moreover, treating the breast cancer cells line MDA-MB-231 with conditioned media containing AP-CXCL14 enhanced cellular proliferation and invasion capacity. Additionally, the proliferative marker Ki67 expression was elevated in the epithelial cells close to the cancer MECs that expressed high CXCL14 in comparison to non-adjacent cells suggesting paracrine effects [75]. It was also reported that elevated expression levels of CXCL12/SDF-1 chemokine and its receptor CXCR4 were associated with increased tumour growth and spread [76, 77]. Consequently, blocking the interaction of CXCL12/CXCR4 using neutralizing antibodies [76] or targeting the expression of CXCR4 via RNAi/CXCR4 [77] suppressed both regional and distant metastasis and prevented tumours growth in vivo [76, 77]. In such a scenario, cancer MECs could contribute to facilitating tumour aggressiveness and metastasis and further destroying the integrity of BM [77].

## Molecular signalling pathways involved in the determination of myoepithelial cells’ fate

### A. Bone morphogenetic protein (BMP) signalling

Luminal and myoepithelial cells are interactively branched during pregnancy yet the precise mechanism that governed the expansion of the MECs components is not fully illustrated. Shao et al., demonstrated the engagement of the bone morphogenetic protein (BMP) signalling pathway in the mammary gland development and morphogenesis and in stabilizing the identity of the MECs during pregnancy [78]. It has been implicated that the BMP pathway involves in controlling the development of different organs including the mammary gland through the regulation of canonical-Smad (a member of the TGF-b superfamily) or non-canonical pathways [79]. The transduction of the BMP signalling cascade is mediated by its receptors BMPR1a, BMPR1b, and BMPR2 [80]. In the mammary gland, BMPR1b was found to facilitate the transition of the mammary stem progenitors into differentiated luminal epithelial cells and hence this could potentially be contributed to tumour formation. On the other hand, interference with BMPR2 receptor expression was associated with tumour advancement and provoked pulmonary metastases in the mice model. BMPR1a receptor is the crucial component in this pathway as in vivo global deletion of Bmpr1a is incompatible with life. During embryonic life, the development and proliferation of the mammary buds are conducted by the effect of the activated BMPR1a in response to PTHrP stimulation [81]. Furthermore, stimulation of BMPR1a delivers numerous biological functions such as growth and proliferation, differentiation and migration, cellular communication, and neural stem cell development [82].

The activation of the BMP pathway is mediated by BMP2/4, the central BMPs pathway components, that binds to BMP receptors (specifically BMPR1a) and transduces the signalling via downstream phosphorylation of Smad-dependant pathway [83]. Activation of Smad signalling includes phosphorylation of Smad 1/5/8 and subsequent interaction with Smad4 that translocate into the nucleus and induces the transcription of different target genes [84, 85]. Under the effect of pregnancy hormones, a significant upregulation of BMPR1a receptors was observed. Activation of BMPR1a downstream BMP signalling by hormone-induced Sp1 resulted in the activation of pSmad1/5-Smad4 complexes and consequently increased the expression of p63 and Slug, the two fundamental regulators of myoepithelial functions and integrity. Furthermore, the conditional knockout (cKO) model of BMPR1a resulted in defective myoepithelial-luminal balanced, loss of myoepithelial integrity, and compromised mammary stem cell population. Interestingly, the BMPR1a cKO mice displayed premature alveolar maturity and differentiation during pregnancy shown by the expansion of lobuloalveolar structures and decrease in the Lin^−^ CD24^+^ CD29^*high*^ myoepithelial cells and an upsurge in the Lin^−^ CD24^+^ CD29^*low*^ luminal cells [78]. Consistently, protein analysis studies revealed reduced levels of the myoepithelial marker CK14 signifying the importance of the activation of the BMPR1a receptors in maintaining MECs differentiation fate [78]. To further validate the critical role provided by BMP signalling in mediating functional differentiated MECs, comparative genes analysis profile via RNA-seq was conducted. BMPR1a cKO model demonstrated a significant reduction in many MECs genes such as *Itgb3, Adamts18, Cdh2, Tspan8, p63, and Slug* compared to the control group [78].

On the other side of the spectrum, treatment of HC11 mammary epithelial cells with BMP4 followed by activation of BMP signalling and the downstream pSmad1/5-Smad4 complex ensued upregulation in p63 and Slug expression [78]. Previous reports have identified the role of p63 and Slug in regulating the differentiation of mammary stem cells into myoepithelial cells. P63, through interacting with WNT signalling, was found to determine the fate of the mammary stem cell progenitors in the differentiated MECs [86]. Furthermore, p63 was involved in the induction of unipotent mammary basal progeny derived from the embryonic multipotent precursors and promoting the transition of luminal cells into basal epithelial cells [87]. Another key regulator of mammary stem cell differentiation into myoepithelial cells is Slug [88]. Expression of Slug is necessary for sustaining the mesenchymal state of the MECs through interaction with LCD1 and impeding the luminal phenotype differentiation [89]. Additionally, in the population of MECs, a lack of Slug expression was linked to a dysfunctional DNA repair pathway [88]. As a result, p63 and Slug are both essential in ensuring the MEC's integrity and good operation. Of note, the formation of precocious alveolar differentiation, which has a phenotype similar to the BMPR1a receptor cKO model, was caused by targeted P-cadherin expression, as previously demonstrated (in the section Anatomical and histogenesis portrayal of the myoepithelial cells) [90, 91]. P-cadherin mRNA expression and protein levels increased in response to BMP4 stimulation of the HC11 mammary epithelium via activation of the BMP cascade. These results confirmed the crucial function of the BMP pathway in maintaining the health and integrity of MECs and preventing early alveolar branching via the BMPR1a/p63/P-cadherin and BMPR1a/Slug/P-cadherin pathways [78]. Moreover, the contribution of the BMP signalling cascade in promoting mammary acini organization was further authenticated in the ex-vivo mammary epithelium model. Using 3D culture analysis, induction of cellular differentiation and encouraging the acinar formation in normal mammary epithelial cells were obtained in response to the administration of BMP4 protein [92].

The participation of the BMP signalling pathway in maintaining adult muscle homeostasis has been evidenced by several other reports [93, 94]. BMP cascade was recently reported to derive muscular hypertrophy in the adult muscle which is speculated to be induced by suppressing the myostatin signalling [93]. Sustained stimulation of type I BMP receptors leads to releasing of Smad4 with its nuclear translocation and consequently induction of muscle hypertrophy [94]. Thus targeting the expression of Smad4 in the mice muscles results in muscle atrophy and wasting [93]. On the other hand, a negative regulator of muscle growth and proliferation in adults is myostatin. Myostatin is one of the TGF-b superfamily members that interact with activin receptors type1 and II causing phosphorylation of Smad2/3 with the subsequent complex formation with Smad4 and induction of gene expression promoting muscle wasting [93].

Metastasis is considered a clinical challenge and has been claimed as a major reason for mortality in breast cancer patients. Therefore, efforts to overcome/limit the metastatic potency of breast cancers have been dedicated. Basal-like triple-negative breast cancers (TNBCs) represent an aggressive subtype with high metastatic capacity harnessed to the enrichment of the cancer stem cell population CD44^high^/CD24^low^. In transcriptome analysis, BMP4 expression was reported to be remarkably suppressed in TNBCs upon TGFβ stimulation [92]. Low Bmp4 gene expression was also found in highly aggressive metastatic murine mammary tumours [95]. Likewise, an IHC study of a tissue microarray from 535 breast cancer patients’ samples displayed low BMP4 protein levels in DCIS and IBC as compared to benign breast tissues thus suggesting a metastasis inhibitor role mediated by BMP4 [96]. Accordingly, in vitro treatment of TNBCs with BMP4 protein, resulted in a significant reduction in cancer stem cell populations [92]. Similarly, a preclinical in vivo animal study showed the ability of BMP4 in preventing breast cancer spontaneous metastases. BMP4, through activation of the BMP-SMAD pathway, modulated the expression of several metastases-associated genes, such as Smad7. Indeed, therapeutic supplementation of recombinant BMP4 protein or restoring the expression of BMP4 sensitized the breast cancer cells to anoikis and significantly decreased the volume of circulating cancer cells with subsequent inhibition of metastatic niches to the bones and lungs [96]. Clinically, in silico data analysis revealed a favourable correlation between BMP4 high expression level and prolong overall survival (OS) and relapse-free survival (RFS) in breast cancer patients. Conversely, poor breast cancer patients’ outcomes in the context of short distant-metastasis–free survival (DMFS), OS, and RFS were associated with low BMP4 expression levels using a multivariate analysis [96].

### B. Myocardin-related transcription factor A (MRTF-A)

It was mentioned earlier that MECs demonstrated phenotypic features of both epithelial cells and smooth muscle cells by expressing different molecular markers related to each group of cells. Myocardin is a protein that is known to be expressed specifically in cardiac and smooth muscle cells. Myocardin has several transcription factors that are widely distributed among different cell types such as MRTF-A/MAL/MKL1 and MRTF-B/MKL-2 [17]. In vivo investigation, targeting the expression of the Myocardin gene or MRTF-B gene causes early embryonic lethality at E10.5 due to precluding of smooth muscle cell differentiation and abnormality in cardiac arteries formation. On the other hand, the MRTF-A gene KO mice model is compatible with life, yet the MRTF-A mutant female mice failed to lactate and nurse their litters, otherwise, no abnormal maternal behaviours were observed.

Compared to the control group, MRTF-A KO mice offspring exhibited retardation in growth rate and early death at 20 days. From the histological analysis of the mammary gland in both groups, overall, no abnormal morphology in the ductal tree branching was detected and the fatty tissues were normally distributed during pregnancy, resting phase, and postpartum periods. Nevertheless, on day 12 of lactation, as compared to the wild type, the mammary gland of the MRTF-A mutant female revealed scarce fully differentiated MECs that were associated with apoptosis of these populations. These findings are translated by the inability of MECs to contract and the failure of milk ejection in the postpartum lactation period. Furthermore, the mammary ductal tree of the KO mice is characterized by disturbed, thinner, pale, and dilated alveolar walls showing trapping of accumulated milk as compared to the wild-type group. These results were further corroborated by protein analysis extracted from the mutant mice during lactation and displaying the absence of smooth muscle proteins responsible for contractile effects including SMA, SM MHC and SM caldesmon. Furthermore, other MECs genes also showed reduced expression levels such as CK14 and CALLA genes and thus explain the failure of milk propelling out of the ductal tree during lactation in the KO mice group [17]. No other morphological abnormalities in the mammary gland in both groups were observed during involution and after weaning of the pups yet a large portion of the dead cells in the mutant female mice group was made of the MECs population. This study concluded the crucial contribution of MRFT-A in ensuring proper maturation and differentiation of MECs and in maintaining their viability and proper contractile functions [17].

### C. Integrin receptors signalling

a3b1 integrin, a laminin receptor, is one of the main mediators of MECs contractility in the mammary gland. Integrins receptors are heterodimeric adhesion molecules that constitute the major components of the mammary epithelium ECM [97]. They that act as a platform for signalling communications by connecting the ECM to the actin filaments of the cytoskeleton within focal adhesion structures attesting to cytoskeleton stabilization. Integrins delivered this stabilization effect via regulating molecules involved in cell–cell and cell-ECM communications such as Rho-GTPases [98]. Integrins can regulate the transduction of different biological signals harnessed to growth, proliferation, survival, differentiation, motility, and cytokeratin’s integrity as well as rheostats the intracellular chemical signals [99, 100].

Regulation of the contraction/relaxation cycle in MECs is attained by myosin light chain phosphorylation (P-MLC) and this is conducted by two main signalling cascades: Oxytocin and a3b1 integrin receptors pathway. The contraction phase is mediated by the binding of oxytocin to its receptors (OT-R) on the MECs yielding activation of two compartments: the RhoA/ROCK cascade with MLCP suppression and the activation of phospholipase/calcium/MLCK signalling with the net outcome of MLC phosphorylation (P-MLC) and subsequent myoepithelial contraction. The contraction cycle of MECs is balanced by stimulation of a3b1 integrin receptors in MECs resulting in activation of FAK/Rac/PAK pathway and MLCK inhibition followed by MLC de-phosphorylation (P-MLC > MLC) and subsequent MECs relaxation [101].

Germline deletion of a3b1 integrin receptors is associated with infant death as a consequence of organ malformations mainly in the lungs and the kidneys [102]. In vivo investigations showed that conditional ablation of a3b1 integrin receptor expression in the MECs using the Cre-Lox strategy resulted in a diminishing in their contractility power with subsequent failure of milk secretion during lactation. Interestingly, the structural differentiation of the mammary gland and alveologenesis was left unchanged. Moreover, immunoblots for milk proteins and contraction-related proteins were the same in both groups. Molecularly, mutant female mice demonstrated an impairment of FAC activation, the imbalance between Rho/Rac pathways, and sustained phosphorylation of MLC associated with the hypercontractile phenotype of MECs. Furthermore, in vitro studies confirmed that the lack of a3b1 integrin receptors in mammary MECs weakens the relaxation cycle yet the cells-maintained contraction upon oxytocin treatment. Additionally, treatment of the mutant cells with MLCK inhibitors or overexpression with activated PAK or Rac rescued the relaxation cycle of the mutant phenotype and prevent additional contractions. Thus substantiating the strong engagement of a3b1 integrin receptors in mediating the proper and complete contraction/relaxation cycle of the MECs and consequently effective lactation [101].

### D. Numb/Numb1 signalling

During mammary gland development, a balance between myoepithelial-epithelial plasticity-promoting and restricting mechanisms should be maintained to ensure precise ductal elongations and alveologenesis [103]. Two key proteins that were found to contribute significantly to shaping the pattern of the MECs and LECs’ growth and morphogenesis during pregnancy and lactation, are Numb and Numb1proteins. These homologous play several prominent roles in defining the cells' destiny, cellular differentiation, and characterization during the maturity of hematopoietic stem cells, neural stem cells, muscle cells, and cancer stem cells [104]. Numb and Numb1 act by inhibiting the Notch signalling pathway, which determines the mammary stem cell self-renewal capacity and LECs fate during the mammary gland development [105, 106]. Also, the natural withholding of the Notch signalling cascade is required for the establishment of normal breast architecture and a lactogenic background that is conducted by activation of the PRL/PRLR/STAT5 signalling [103].

The MECs revealed high expressed levels of Numb and Numb1proteins and their expression are further upregulated during pregnancy. As compared to the wild type, the conditional KO (cKO) model of Numb and Numb1 showed dilated lumen with a momentous defect in MECs propagation and a significant reduction in SMA + cells, the main marker for effective contractile competency [103]. Additionally, the cKO mice unveiled outgrowth and infiltrating patterns of the LECs associated with precluding the rightful alveologenesis. Furthermore, loss of Numb/Numb1 resulted in the development of mesenchymal phenotype demonstrated by upregulation in EMT markers such as Snail, Slug, Twist, Zeb1, and reduction in the E-cadherin expression [103]. Consistently, these cKO mice were unable to breastfeed their pups due to a lack of milk production along with a hindrance in the MECs’ contractile activity to release the milk to the exterior.

These observed outcome effects in the cKO mice have resulted from the activation of the Notch signalling cascade. Sustained stimulation of Notch signalling increased the expansion of the undifferentiated luminal progenitors’ cells presented by elevated CK8 + cells and decreased CK14 + cell populations. On the other hand, in vitro overexpression of Numb1 blocks cellular migration and inhibits the expression of the EMT invasive markers. Altogether, these findings proved the appreciated engagement of MECs, by expressing Numb/Numb1 proteins, in orchestrating normal mammary gland structure to meet the delivery of competent functions [103].

### E. Inhibitors of differentiation proteins 4 (ID4)

Inhibitors of differentiation proteins 4 (ID4) are transcriptional regulators that have helix-loop-helix structures and are deficient in DNA- binding motifs. They regulate the transcription of different genes by binding to the basic HLH (bHLH) transcription factor protein HEB. Next, this complex binds to the E-box region on the response elements of the regulated genes that contributed to ECM synthesis and cytoskeleton regulatory functions, differentiation, and stemness in different cellular lineages [107]. As its name implied, the ID4 full repertoire of transcriptional targets is involved in the upregulation of the expression of the proliferative genes and downregulation of the expression of the differentiation genes specifically for MECs populations.

The expression of ID4 is restricted to basal cells of the mammary epithelium presenting a fundamental role in mammary stem cell propagation and ductal tree expansion during puberty [108]. Thus, explaining the highest expression level of ID4 in the epithelial cap cells as compared to its lower level in the fully differentiated MECs. Furthermore, ID4 was reported to act as a negative regulator of both luminal [109] and MECs lineage differentiation hierarchy in both in vitro and in vivo investigations [109–112]. Consequently, low ID4 levels during pregnancy and lactation permit the differentiation and specialization of the basal cells into mature and functionally contractile MECs [110]. Indeed, ID4 expression is inversely correlated with the expression of other MECs contractile genes including a-SMA, Cnn1, Cav1, Mylk, Lmod1, Acta2, and Myh11. These findings indicate the involvement of ID4 in inhibiting the expression of the differentiation myogenesis genes. This role of ID4 in blocking luminal and myoepithelial commitments of basal cells would maintain the full identity of the stem cell population during the mammary gland evolution [110].

Of note, ID4 was reported to be engaged in the pathogenesis and progression of breast cancer [113, 114] particularly basal-like breast cancer that enriches in stem cells population providing inferior prognosis [115]. Indeed, expression of ID4 was found to be elevated in aggressive breast cancer human tissue mainly the TNBC and HER2 enriched samples as compared to the normal breast epithelium. Moreover, the high ID4 expression level was associated with advanced tumour pathological staging and grading and correlated with poor patients' clinical outcomes [116]. One report showed that in vitro inhibition of ID4 expression in MCF-7 breast cancer cells following magnetothermal therapy (MTT) using specialized nanoparticles produced a potent reduction in cell viability. These findings were further validated in an animal model where the introduction of siRNA-ID4 nanoparticles into breast cancer xenografted nude mice suppressed tumour growth and reduced its volume by > 98% [117].

## Conclusion

**Fig. 3:**
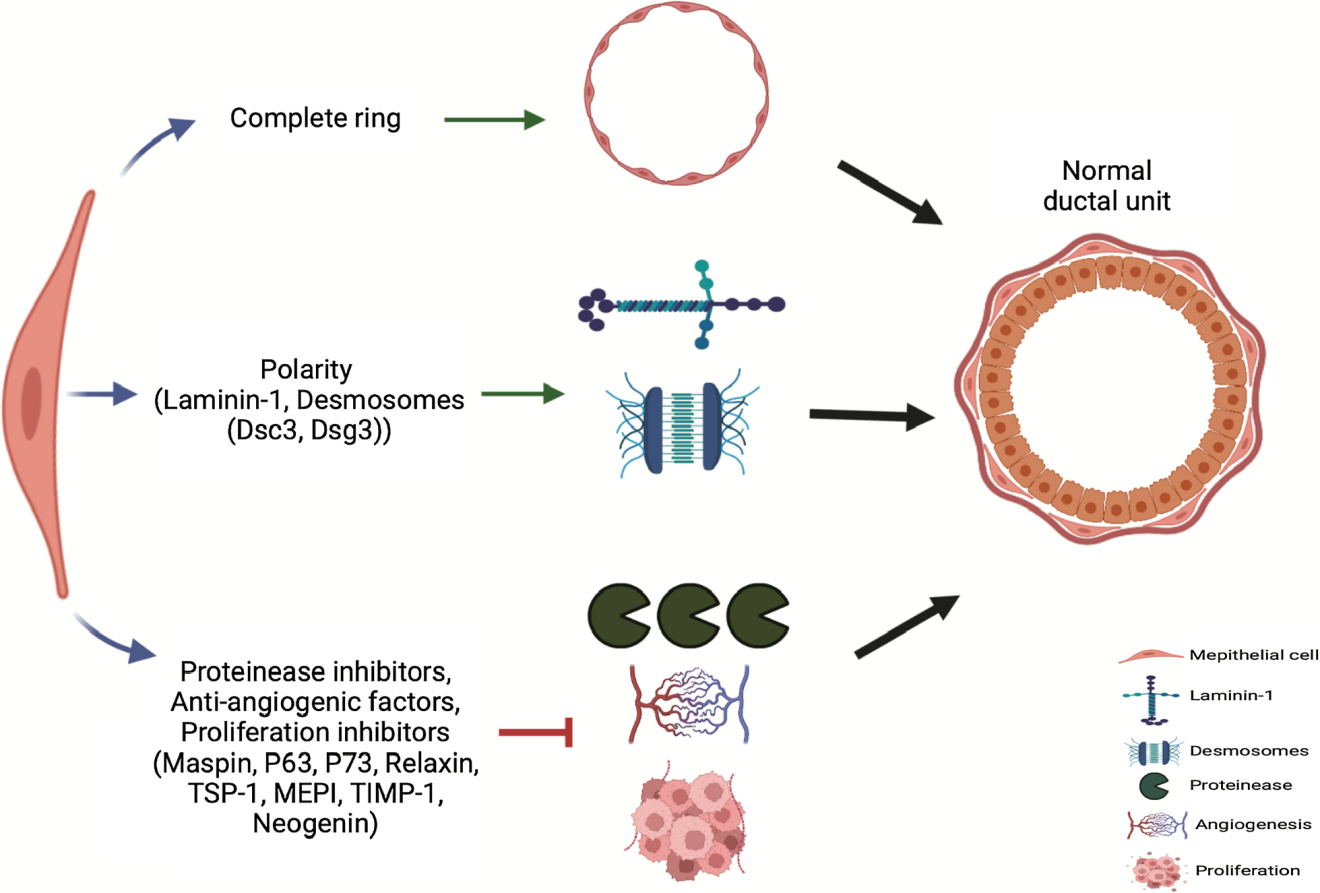
Schematic demonstration of the tumour suppression roles exhibited by the myoepithelial cells: myoepithelial cells are arranged in an integrated ring making a solid fence separating the inner cellular environment away from the surrounding stroma. Myoepithelial cells producing proteins contributed to cellular polarity and organization, Laminin-1 and Desmosomes. Many proteinase inhibitors, ant-angiogenic factors, and growth suppressors are also produced by myoepithelial cells defining the anti-invasive and anti-proliferative capacities owned by this population. *TSP-1* Thrombospondin-1, *MEPI* Myoepithelium-derived serine proteinase inhibitor, and *TIMP-1* Tissue inhibitors of metalloproteinases. Created with BioRender.com

It is postulated and generally accepted that loss of normal cell architecture, organization, and polarity is one of the hallmarks of breast cancer advancement [118]. During breast cancer progression, there is an outnumbered of MECs with a significant increase in LECs proliferation and growth, accordingly, invasive breast carcinomas are exemplified by the complete loss of MECs layers [119]. The MECs layer has gained importance as a guardian of ‘normalcy,’ by forming a barrier partitioning the precancerous proliferating luminal cells away from the surrounding stroma thus deterring the local invasion [35, 67]. Moreover, MECs delivered a paracrine fashion in suppressing cancer progression by leading cellular polarization and producing antiangiogenic factors and proteinase inhibitors, (Fig. 3). These biological features provide a plausible explanation for a poor prognosis of breast carcinomas that exhibited partial or complete defects in the functionally differentiated MECs [120]. In this context, the presence of functional MECs is inevitable to restrain the progression of the malignant cells and limit the conversion of non-invasive tumours to invasive ones [121, 122].

## Outlook

Inducing cancer cell differentiation as a therapeutic modality has been known for decades and showed fruitful results in curing hematological malignancies such as Leukemias [123–125]. This powerful system provided pleasant outcomes with trivial adverse effects in contrast to the traditional cancer cytotoxic treatment including chemo/radiotherapy and anti-endocrine agents. Thereby, stimulating and maintaining of differentiation cellular pathways in breast cancer would offer a promising therapeutic strategy for defeating cancer evolution. Consequently, significant efforts are in need to ascertain regulators and mechanisms of normal/cancer cell differentiation to help in the cessation of advanced disease in breast cancer patients. Herein, we sought to elucidate that full gaining of MECs differentiation is required for their anti-tumorigenic role and the conveyance of proper functions and anticancer guarding mediators. For better persistence of the tumours’ inhibitory effects of the differentiated MECs, we need to unravel which components along the myoepithelial pathways are crucial to deciding on the differentiation fate of the breast cancer cells to a less devastating cell type. Furthermore, an inclusive understanding of the crosstalk between the major signalling pathways regulating the MECs’ destiny is a prerequisite to facilitating the innovation of efficacious therapeutic targets in the field of breast cancer. It is evident that the function of the MECs in the mammary gland extends beyond contractility and thus dissecting the roles of the MECs in both physiological and pathological conditions merits further investigations.

In this study, we depicted many molecular mechanisms, as previously demonstrated, that are involved in the determination of myoepithelial cells’ destiny. BMP via BMP4/BMPR1a accounts for the most imperative signalling cascade in deriving mammary cellular differentiation and shaping the precise identity and integrity of MECs within breast tissues. Also, the discussed studies corroborated the metastatic/invasive suppressor role of BMP4 and defined BMP4 as a favourable prognostic marker in breast cancer patients. On the other hand, the transcriptional factor, ID4, negatively regulates the MECs and LECs differentiation and enhances stem cell enrichment. The anti-differentiation role of ID4 allowed its participation in the evolution of aggressive basal-like breast cancers that are associated with advanced disease and unfavourable patients' prognoses. Therefore, targeting ID4 expression would ensure the continuation of both MECs and LECs’ differentiated phenotype and the production of natural endogenous anti-tumorigenic forces. This shed the light on a tempting therapeutic avenue in treating breast cancer patients. Both BMP4 and ID4 can be proposed as valuable attractive targets in combating metastatic breast cancer disease via promoting proper cellular differentiation hierarchy and exact physiological activities.

## Data Availability

The datasets used during the current study are available in this published form.

## Abbreviations

LECs: Luminal epithelial cells
MECs: Myoepithelial cells
BM: Basement membrane
TDLU: Terminal ducts lobular units
MMPs: Matrix metalloproteinases
*α*-SMA: *α*-Smooth muscle actin
DCIS: Ductal carcinoma in situ
IBC: Invasive breast cancer
MGA: Micro-glandular Adenosis
CAFs: Cancer-associated fibroblasts
lrECM: Laminin-rich extracellular matrix
Dsc 3: Desmocollin 3
Dsg 3: Desmoglein 3
PAS: Plasminogen activation system
VEGF: Vascular endothelial growth factor
NO: Nitric oxide
TSP-1: Thrombospondin-1
TEBs: Terminal end buds
MEPI: Myoepithelium-derived serine proteinase inhibitor
TIMPs: Tissue inhibitors of metalloproteinases
BMP: Bone morphogenetic protein
TNBCs: Triple-negative breast cancers
OS: Overall survival
RFS: Relapse-free survival
DMFS: Distant-metastasis–free survival
MRTF-A: Myocardin-related transcription factor A
ID4: Inhibitors of differentiation proteins 4
MTT: Magnetothermal therapy
EMT: Epithelial-to-mesenchymal transition

## References

[1] Biswas SK (2022). The mammary gland: basic structure and molecular signaling during development. Int J Mol Sci.

[2] Pandey PR, Saidou J, Watabe K (2010). Role of myoepithelial cells in breast tumor progression. Front Biosci.

[3] Deugnier M-A (2002). The importance of being a myoepithelial cell. Breast Cancer Res.

[4] Kolar Z (2007). A novel myoepithelial/progenitor cell marker in the breast?. Virchows Arch.

[5] Sternlicht MD (1997). The human myoepithelial cell is a natural tumor suppressor. Clin Cancer Res: an official journal of the American Association for Cancer Research.

[6] Lakhani SR, O'Hare MJ (2000). The mammary myoepithelial cell-Cinderella or ugly sister?. Breast Cancer Res.

[7] Schnitt SJ (2009). The transition from ductal carcinoma in situto invasive breast cancer: the other side of the coin. Breast Cancer Res..

[8] Winer A, Adams S, Mignatti P (2018). Matrix metalloproteinase inhibitors in cancer therapy: turning past failures into future successes. Mol Cancer Ther.

[9] Kapoor C (2016). Seesaw of matrix metalloproteinases (MMPs). J Cancer Res Ther.

[10] Mitchell E (2020). Loss of myoepithelial calponin-1 characterizes high-risk ductal carcinoma in situ cases, which are further stratified by T cell composition. Mol Carcinog.

[11] Man Y-G (2007). Focal degeneration of aged or injured myoepithelial cells and the resultant auto-immunoreactions are trigger factors for breast tumor invasion. Med Hypotheses.

[12] Schnitt SJ (2009). The transition from ductal carcinoma in situto invasive breast cancer: the other side of the coin. Breast Cancer Res.

[13] Man Y-G, Sang Q-XA (2004). The significance of focal myoepithelial cell layer disruptions in human breast tumor invasion: a paradigm shift from the “protease-centered” hypothesis. Exp Cell Res.

[14] Gudjonsson T (2005). Myoepithelial cells: their origin and function in breast morphogenesis and neoplasia. J Mammary Gland Biol Neoplasia.

[15] Adriance MC (2005). Myoepithelial cells: good fences make good neighbors. Breast Cancer Res.

[16] Yang J (2016). Overexpressed genes associated with hormones in terminal ductal lobular units identified by global transcriptome analysis: an insight into the anatomic origin of breast cancer. Oncol Rep.

[17] Li S (2006). Requirement of a myocardin-related transcription factor for development of mammary myoepithelial cells. Mol Cell Biol.

[18] Moumen M (2011). The mammary myoepithelial cell. Int J Dev Biol.

[19] Reversi A, Cassoni P, Chini B (2005). Oxytocin receptor signaling in myoepithelial and cancer cells. J Mammary Gland Biol Neoplasia.

[20] Koukoulis G (1991). Immunohistochemical localization of integrins in the normal, hyperplastic, and neoplastic breast. Correlations with their functions as receptors and cell adhesion molecules. Am J Pathol.

[21] Muschler J, Streuli CH (2010). Cell–matrix interactions in mammary gland development and breast cancer. Cold Spring Harb Perspect Biol.

[22] Jones J (2003). Primary breast myoepithelial cells exert an invasion-suppressor effect on breast cancer cells via paracrine down-regulation of MMP expression in fibroblasts and tumour cells. J Pathol: A Journal of the Pathological Society of Great Britain and Ireland.

[23] Boecker W, Buerger H (2003). Evidence of progenitor cells of glandular and myoepithelial cell lineages in the human adult female breast epithelium: a new progenitor (adult stem) cell concept. Cell Prolif.

[24] Petersen OW, van Deurs B (1988). Growth factor control of myoepithelial-cell differentiation in cultures of human mammary gland. Differentiation.

[25] Haaksma CJ, Schwartz RJ, Tomasek JJ (2011). Myoepithelial cell contraction and milk ejection are impaired in mammary glands of mice lacking smooth muscle alpha-actin. Biol Reprod.

[26] Jolicoeur F (2005). Intrauterine breast development and the mammary myoepithelial lineage. J Mammary Gland Biol Neoplasia.

[27] Jin R (2001). Significance of metallothionein expression in breast myoepithelial cells. Cell Tissue Res.

[28] Sternlicht M, Barsky S (1997). The myoepithelial defense: a host defense against cancer. Med Hypotheses.

[29] Yu GH (1997). Benign pairs. A useful discriminating feature in fine needle aspirates of the breast. Acta cytological..

[30] Gusterson BA (1982). Distribution of myoepithelial cells and basement membrane proteins in the normal breast and in benign and malignant breast diseases. Can Res.

[31] Virnig BA (2009). Diagnosis and management of ductal carcinoma in situ (DCIS). Evid Rep Technol Assess.

[32] Clark S (2011). Molecular subtyping of DCIS: heterogeneity of breast cancer reflected in pre-invasive disease. Br J Cancer.

[33] Barsky SH, Karlin NJ (2005). Myoepithelial cells: autocrine and paracrine suppressors of breast cancer progression. J Mammary Gland Biol Neoplasia.

[34] Carter EP (2017). A 3D in vitro model of the human breast duct: a method to unravel myoepithelial-luminal interactions in the progression of breast cancer. Breast Cancer Res.

[35] Rakha EA (2018). Invasion in breast lesions: the role of the epithelial–stroma barrier. Histopathology.

[36] Risom T (2022). Transition to invasive breast cancer is associated with progressive changes in the structure and composition of tumor stroma. Cell.

[37] Friedman G (2020). Cancer-associated fibroblast compositions change with breast cancer progression linking the ratio of S100A4+ and PDPN+ CAFs to clinical outcome. Nature Cancer.

[38] Chatterjee SJ, McCaffrey L (2014). Emerging role of cell polarity proteins in breast cancer progression and metastasis. Breast Cancer (Dove Med Press).

[39] Halaoui R (2017). Progressive polarity loss and luminal collapse disrupt tissue organization in carcinoma. Genes Dev.

[40] Catterall R, Lelarge V, McCaffrey L (2020). Genetic alterations of epithelial polarity genes are associated with loss of polarity in invasive breast cancer. Int J Cancer.

[41] Zhao Y (2021). Loss of polarity protein Par3 is mediated by transcription factor Sp1 in breast cancer. Biochem Biophys Res Commun.

[42] Li J (2014). Loss of LKB1 disrupts breast epithelial cell polarity and promotes breast cancer metastasis and invasion. J Exp Clin Cancer Res.

[43] Gudjonsson T (2002). Normal and tumor-derived myoepithelial cells differ in their ability to interact with luminal breast epithelial cells for polarity and basement membrane deposition. J Cell Sci.

[44] Zou Z (1994). Maspin, a serpin with tumor-suppressing activity in human mammary epithelial cells. Science.

[45] Runswick SK (2001). Desmosomal adhesion regulates epithelial morphogenesis and cell positioning. Nat Cell Biol.

[46] Bissell MJ, Bilder D (2003). Polarity determination in breast tissue: desmosomal adhesion, myoepithelial cells, and laminin 1. Breast Cancer Res.

[47] Carlisle JW, Harvey RD (2021). Tyrosine kinase inhibitors, antibody-drug conjugates, and proteolysis-targeting chimeras: the pharmacology of cutting-edge lung cancer therapies. Am Soc Clin Oncol Educ Book.

[48] Wyganowska-Świątkowska M (2019). Proteolysis is the most fundamental property of malignancy and its inhibition may be used therapeutically (Review). Int J Mol Med.

[49] Radisky ES, Raeeszadeh-Sarmazdeh M, Radisky DC (2017). Therapeutic potential of matrix metalloproteinase inhibition in breast cancer. J Cell Biochem.

[50] Abou Shousha SA (2022). Angiogenic activities of interleukin-8, vascular endothelial growth factor and matrix metalloproteinase-9 in breast cancer. Egypt J Immunol.

[51] Xiao JP (2005). Relation between angiogenesis, fibrinolysis and invasion/metastasis in breast cancer. Zhonghua Zhong Liu Za Zhi.

[52] *[Expert consensus on off-label use of small molecule anti-angiogenic drugs in the treatment of metastatic breast cancer].* Zhonghua Zhong Liu Za Zhi, 2022. **44**(6): 523–530. 10.3760/cma.j.cn112152-20220310-00168 35754226

[53] Foschini MP, Eusebi V (1998). Carcinomas of the breast showing myoepithelial cell differentiation. Virchows Arch.

[54] Angele S (2004). Expression of ATM, p53, and the MRE11–Rad50–NBS1 complex in myoepithelial cells from benign and malignant proliferations of the breast. J Clin Pathol.

[55] Barsky SH (2003). Myoepithelial mRNA expression profiling reveals a common tumor-suppressor phenotype. Exp Mol Pathol.

[56] Sternlicht MD (1996). Characterizations of the extracellular matrix and proteinase inhibitor content of human myoepithelial tumors. Lab Invest: a journal of technical methods and pathology.

[57] Nguyen M (2000). The human myoepithelial cell displays a multifaceted anti-angiogenic phenotype. Oncogene.

[58] Zhang M (2000). Volpert 0, Shi YH and Bouck N: Maspin is an angiogenesis inhibitor. Nat Med.

[59] Pemberton PA (1995). The tumor suppressor maspin does not undergo the stressed to relaxed transition or inhibit trypsin-like serine proteases: evidence that Maspin is not a protease inhibitory serpin (∗). J Biol Chemis.

[60] Zhang RR (2003). A subset of morphologically distinct mammary myoepithelial cells lacks corresponding immunophenotypic markers. Breast Cancer Res.

[61] Simpson PT (2004). Distribution and significance of 14-3-3σ, a novel myoepithelial marker, in normal, benign, and malignant breast tissue. J Pathol.

[62] Yamamoto T (2001). p73 is highly expressed in myoepithelial cells and in carcinomas with metaplasia. Int J Oncol.

[63] Bani D (1995). Relaxin activates the L-arginine-nitric oxide pathway in human breast cancer cells. Can Res.

[64] Xiao G (1999). Suppression of breast cancer growth and metastasis by a serpin myoepithelium-derived serine proteinase inhibitor expressed in the mammary myoepithelial cells. Proc Natl Acad Sci U S A.

[65] Keeling S, Gad J, Cooper H (1997). Mouse Neogenin, a DCC-like molecule, has four splice variants and is expressed widely in the adult mouse and during embryogenesis. Oncogene.

[66] Brew K, Dinakarpandian D, Nagase H (2000). Tissue inhibitors of metalloproteinases: evolution, structure and function. Biochimica et Biophysica Acta.

[67] Sirka OK, Shamir ER, Ewald AJ (2018). Myoepithelial cells are a dynamic barrier to epithelial dissemination. J Cell Biol.

[68] Cerchiari AE (2015). A strategy for tissue self-organization that is robust to cellular heterogeneity and plasticity. Proc Natl Acad Sci U S A.

[69] Maître JL (2012). Adhesion functions in cell sorting by mechanically coupling the cortices of adhering cells. Science.

[70] Grudzien-Nogalska E, Reed BC, Rhoads RE (2014). CPEB1 promotes differentiation and suppresses EMT in mammary epithelial cells. J Cell Sci.

[71] Shao Z-M (1998). The human myoepithelial cell exerts antiproliferative effects on breast carcinoma cells characterized by p21WAF1/CIP1Induction, G2/M Arrest, and Apoptosis. Exp Cell Res.

[72] Barsky SH, Karlin NJ (2006). Mechanisms of disease: breast tumor pathogenesis and the role of the myoepithelial cell. Nat Clin Pract Oncol.

[73] Masih M (2022). Role of chemokines in breast cancer. Cytokine.

[74] Hall JM, Korach KS (2003). Stromal cell-derived factor 1, a novel target of estrogen receptor action, mediates the mitogenic effects of estradiol in ovarian and breast cancer cells. Mol Endocrinol.

[75] Allinen M (2004). Molecular characterization of the tumor microenvironment in breast cancer. Cancer Cell.

[76] Müller A (2001). Involvement of chemokine receptors in breast cancer metastasis. Nature.

[77] Smith MC (2004). CXCR4 regulates growth of both primary and metastatic breast cancer. Can Res.

[78] Shao C (2021). Hormone-responsive BMP signaling expands myoepithelial cell lineages and prevents alveolar precocity in mammary gland. Front Cell and Dev Biol.

[79] Heldin C-H, Miyazono K, Ten Dijke P (1997). TGF-β signalling from cell membrane to nucleus through SMAD proteins. Nature.

[80] Horbelt D, Denkis A, Knaus P (2012). A portrait of Transforming Growth Factor β superfamily signalling: background matters. Int J Biochem Cell Biol.

[81] Robinson GW (2007). Cooperation of signalling pathways in embryonic mammary gland development. Nat Rev Genet.

[82] Wegleiter T (2019). Palmitoylation of BMPR1a regulates neural stem cell fate. Proc Natl Acad Sci.

[83] Reise SP, Waller NG (2009). Item response theory and clinical measurement. Annu Rev Clin Psychol.

[84] Qi Z (2017). BMP restricts stemness of intestinal Lgr5+ stem cells by directly suppressing their signature genes. Nat Commun.

[85] Derynck R, Zhang YE (2003). Smad-dependent and Smad-independent pathways in TGF-β family signalling. Nature.

[86] Ding L (2019). Perturbed myoepithelial cell differentiation in BRCA mutation carriers and in ductal carcinoma in situ. Nat Commun.

[87] Wuidart A (2018). Early lineage segregation of multipotent embryonic mammary gland progenitors. Nat Cell Biol.

[88] Gross KM (2019). Loss of slug compromises DNA damage repair and accelerates stem cell aging in mammary epithelium. Cell Rep.

[89] Phillips S (2014). Cell-state transitions regulated by SLUG are critical for tissue regeneration and tumor initiation. Stem cell Rep.

[90] Albergaria A (2011). P-cadherin role in normal breast development and cancer. Int J Dev Biol.

[91] Radice GL (1997). Precocious mammary gland development in P-cadherin–deficient mice. J Cell Biol.

[92] Yan G (2021). TGFβ/cyclin D1/Smad-mediated inhibition of BMP4 promotes breast cancer stem cell self-renewal activity. Oncogenesis.

[93] Sartori R (2013). BMP signaling controls muscle mass. Nat Genet.

[94] Winbanks CE (2013). The bone morphogenetic protein axis is a positive regulator of skeletal muscle mass. J Cell Biol.

[95] Bidwell BN (2012). Silencing of Irf7 pathways in breast cancer cells promotes bone metastasis through immune escape. Nat Med.

[96] Eckhardt BL (2020). Activation of canonical BMP4-SMAD7 signaling suppresses breast cancer metastasis. Cancer Res.

[97] Taddei I (2003). Integrins in mammary gland development and differentiation of mammary epithelium. J Mammary Gland Biol Neoplasia.

[98] Hynes RO (2002). Integrins: bidirectional, allosteric signaling machines. Cell.

[99] Glukhova MA, Streuli CH (2013). How integrins control breast biology. Curr Opin Cell Biol.

[100] Vicente-Manzanares M, Choi CK, Horwitz AR (2009). Integrins in cell migration–the actin connection. J Cell Sci.

[101] Raymond K (2011). Control of mammary myoepithelial cell contractile function by α3β1 integrin signalling. EMBO J.

[102] Kreidberg JA (1996). Alpha 3 beta 1 integrin has a crucial role in kidney and lung organogenesis. Development.

[103] Zhang Y (2016). Numb and Numbl act to determine mammary myoepithelial cell fate, maintain epithelial identity, and support lactogenesis. FASEB J.

[104] Gulino A, Di Marcotullio L, Screpanti I (2010). The multiple functions of Numb. Exp Cell Res.

[105] Beres BJ (2011). Numb regulates Notch1, but not Notch3, during myogenesis. Mech Dev.

[106] Bray SJ (2006). Notch signalling: a simple pathway becomes complex. Nat Rev Mol Cell Biol.

[107] Massari ME, Murre C (2000). Helix-loop-helix proteins: regulators of transcription in eucaryotic organisms. Mol Cell Biol.

[108] Lim E (2010). Transcriptome analyses of mouse and human mammary cell subpopulations reveal multiple conserved genes and pathways. Breast Cancer Res.

[109] Best SA (2014). Dual roles for Id4 in the regulation of estrogen signaling in the mammary gland and ovary. Development.

[110] Holliday H (2021). Inhibitor of Differentiation 4 (ID4) represses mammary myoepithelial differentiation via inhibition of HEB. Iscience.

[111] Baker LA, Holliday H, Swarbrick A (2016). ID4 controls luminal lineage commitment in normal mammary epithelium and inhibits BRCA1 function in basal-like breast cancer. Endocr Relat Cancer.

[112] Junankar S (2015). ID4 controls mammary stem cells and marks breast cancers with a stem cell-like phenotype. Nat Commun.

[113] Donzelli S (2018). Expression of ID4 protein in breast cancer cells induces reprogramming of tumour-associated macrophages. Breast Cancer Res.

[114] Zhang X (2020). ID4 promotes breast cancer chemotherapy resistance via CBF1-MRP1 Pathway. J Cancer.

[115] Junankar S (2015). ID4 controls mammary stem cells and marks breast cancers with a stem cell-like phenotype. Nat Commun.

[116] Garcia-Escolano M (2021). ID1 and ID4 are biomarkers of tumor aggressiveness and poor outcome in immunophenotypes of breast cancer. Cancers (Basel).

[117] Dai P (2021). Regulation of ID4 in vivo for efficient magnetothermal therapy of breast cancer. Advanced Therapeutics.

[118] Kasami M (1998). Maintenance of polarity and a dual cell population in adenoid cystic carcinoma of the breast: an immunohistochemical study. Histopathology.

[119] Rudland PS (1987). Stem cells and the development of mammary cancers in experimental rats and in humans. Cancer Metastasis Rev.

[120] Malzahn K (1998). Biological and prognostic significance of stratified epithelial cytokeratins in infiltrating ductal breast carcinomas. Virchows Arch.

[121] Kenny PA, Bissell MJ (2003). Tumor reversion: correction of malignant behavior by microenvironmental cues. Int J Cancer.

[122] Bissell, M., P. Kenny, and D. Radisky. *Microenvironmental regulators of tissue structure and function also regulate tumor induction and progression: the role of extracellular matrix and its degrading enzymes*. in *Cold Spring Harbor symposia on quantitative biology*. 2005. Cold Spring Harbor Laboratory Press. 10.1101/sqb.2005.70.013 PMC3004779 16869771

[123] Xu WP, Zhang X, Xie WF (2014). Differentiation therapy for solid tumors. J Dig Dis.

[124] Burnett AK (2015). Arsenic trioxide and all-trans retinoic acid treatment for acute promyelocytic leukaemia in all risk groups (AML17): results of a randomised, controlled, phase 3 trial. Lancet Oncol.

[125] de Thé H (2018). Differentiation therapy revisited. Nat Rev Cancer.

[126] Kai K, et al. Breast cancer stem cells. Breast Cancer. 2010;17(2):80–85. 10.1007/s12282-009-0176-y 19806428

